# Whitefly Genome Expression Reveals Host-Symbiont Interaction in Amino Acid Biosynthesis

**DOI:** 10.1371/journal.pone.0126751

**Published:** 2015-05-22

**Authors:** Santosh Kumar Upadhyay, Shailesh Sharma, Harpal Singh, Sameer Dixit, Jitesh Kumar, Praveen C Verma, K. Chandrashekar

**Affiliations:** 1 Department of Botany, Panjab University, Chandigarh, 160014, India; 2 National Agri-Food Biotechnology Institute, (Department of Biotechnology, Government of India), C-127, Industrial Area, S.A.S. Nagar, Phase 8, Mohali, Punjab, 160071, India; 3 CSIR-National Botanical Research Institute, Council of Scientific and Industrial Research, Rana Pratap Marg, Lucknow, 226001, India; 4 Indian Agricultural Research Institute-Regional Station, Baner phata, ITI road, Aundh, Pune, 411007, Maharashtra, India; 5 Academy of Scientific and Innovative Research (AcSIR), Anusandhan Bhawan, 2-Rafi Marg, New Delhi, India; Volcani Center, ISRAEL

## Abstract

**Background:**

Whitefly (*Bemisia tabaci*) complex is a serious insect pest of several crop plants worldwide. It comprises several morphologically indistinguishable species, however very little is known about their genetic divergence and biosynthetic pathways. In the present study, we performed transcriptome sequencing of Asia 1 species of *B*. *tabaci* complex and analyzed the interaction of host-symbiont genes in amino acid biosynthetic pathways.

**Methodology/Principal Findings:**

We obtained about 83 million reads using Illumina sequencing that assembled into 72716 unitigs. A total of 21129 unitigs were annotated at stringent parameters. Annotated unitigs were mapped to 52847 gene ontology (GO) terms and 131 Kyoto encyclopedia of genes and genomes (KEGG) pathways. Expression analysis of the genes involved in amino acid biosynthesis pathways revealed the complementation between whitefly and its symbiont partner *Candidatus Portiera aleyrodidarum*. Most of the non-essential amino acids and intermediates of essential amino acid pathways were supplied by the host insect to its symbiont. The symbiont expressed the pathways for the essential amino acids arginine, threonine and tryptophan and the immediate precursors of valine, leucine, isoleucine and phenyl-alanine. High level expression of the amino acid transporters in the whitefly suggested the molecular mechanisms for the exchange of amino acids between the host and the symbiont.

**Conclusions/Significance:**

Our study provides a comprehensive transcriptome data for Asia 1 species of *B*. *tabaci* complex that focusses light on integration of host and symbiont genes in amino acid biosynthesis pathways.

## Introduction

Whitefly (*Bemisia tabaci*) complex is one of the most vicious insect pests of field crops worldwide. They attack more than 600 species of plants and cause direct (by phloem feeding) as well as indirect (by virus transmission) damages to crops. They also secrete honeydew on plant parts which promotes the growth of fungi [1–3]. At least 24 morphologically indistinguishable biotypes are known in *B*. *tabaci* complex, which are categorized into 11 groups/species [4]. These differ in genetic composition, mating behaviour, fecundity, host range, isozymes, virus transmission ability etc. [5,6]. Transcriptome analysis of three different species MEAM 1, MED and Asia II3 indicated significant divergence in their genetic composition [7–9]. Additionally the genomic data of other species is also required to resolve further divergence, and analyse imperative biochemical pathways in whitefly.

Whiteflies depend upon the phloem sap as a food source. Since phloem sap is deficient in several essential amino acids [10], these insects harbor microbial endosymbionts to complement the requirement of these amino acids [11]. In return, the host insect provides the non-essential amino acids to the symbionts [12]. Symbiosis of *Buchnera aphidicola* and *Acyrthosiphon pisum* has been studied in some details [11] but not so far in whiteflies. The genome sequence of *Candidatus Portiera aleyrodidarum* (CPA, an endosymbiont of whiteflies) and transcriptome data of whiteflies has recently been availed that can be precisely used for analysing the host-symbiont interaction with respect to the amino acid biosynthesis [9, 13]. The presence of all the genes required for the synthesis of essential amino acids like threonine and tryptophan has been noticed in CPA by genome sequencing. The last genes for the biosynthesis of valine, leucine, isoleucine and phenylalanine are not found in CPA. Genes involved in the synthesis of most of the nonessential amino acids are completely absent in the CPA genome [13]. However, the above analysis is solely based on the presence of genes in the genome, and has not been validated at the transcriptome and protein levels. Genes related to the transporters of amino acids and other metabolites across the host and the symbiont are also important in facilitating their cooperation but have not been explored in the present study.

However, in the present study, we performed comprehensive transcriptome sequencing of Asia 1 species of *B*. *tabaci* [4] and analysed the cooperation of host and symbiont genes in amino acid biosynthesis and transportation. Based on origin, function annotation, biosynthetic pathway mapping and expression of transcripts, we proposed interaction between whitefly and its endosymbiont CPA genes in amino acid biosynthesis.

## Materials and Methods

### Insect rearing

The culture of whitefly (*B*. *tabaci* Asia 1 species) was maintained on cotton (*Gossypium hirsutum*) plants in controlled environmental conditions as described earlier [14]. The purity of the culture was analyzed by sequencing of cytochrome oxidase I (COI, GenBank accession no: KJ864965) and ITS1 genes as reported earlier [4, 15–16]. In order to get comprehensive transcriptome data, samples from different developmental stages (egg, nymph, pupa and adult) of insects were collected. Eggs and nymphs were collected as one sample due to their small sizes, and pupa and adults were collected separately. These samples were frozen in liquid nitrogen and stored at -80°C till further use.

### RNA isolation, sample preparation and Illumina sequencing

Total RNA was isolated from each sample separately using Tri reagent (Sigma, USA), following the manufacturer’s protocol. DNA contamination in the RNA preparations was removed by DNA-free Kit (Ambion, USA). The integrity of the RNA samples was analysed on the 2100 Bioanalyzer (Agilent Technologies, USA). Equal amount (5 μg) of total RNA from each sample was pooled, and mRNA was purified once to retain the symbionts transcripts as described [17]. The mRNA was fragmented and cDNA library was prepared using ‘Tru Seq RNA sample preparation kit v2 from Illumina following the manufacturer’s protocol (http://www.illumina.com/systems/hiseqsystems/hiseq20001000/kits.ilmn). The library was sequenced, in a Paired End 100 base run, using TruSeq PE Cluster Kit v3-cBot-HS for cluster generation on C-bot and TruSeq SBS Kit v3-HS for sequencing, on the Illumina HiSeq1000 platform following the standard protocols provided by the manufacturer. Illumina sequencing data is available at the NCBI Short Read Archive (SRA) database (accession number: SRR1159208).

### Assembly of Illumina sequencing reads

Adapter sequences, low quality reads (reads containing ambiguous sequences ‘N’) and empty reads were removed from raw sequencing data. The reads were assembled into scaffolds using ABySS (Assembly By Short Sequences) paired end assembly program (http://www.bcgsc.ca/platform/bioinfo/software/abyss) at k-mer 21 [18], which gave the best assembly result. Scaffolds were further assembled by CAP3 (contig assembly program) at a high stringent parameter of minimum 40 bp overlap with more that 95% sequence similarity [19]. Contigs and singletons obtained after CAP3 assembly were pooled together and used as distinct sequences for further characterization. Assembled sequences had been already deposited as Transcriptome Shotgun Assembly projects at DDBJ/EMBL/GenBank under the accession numbers GAUC00000000 and GAUL00000000. The version described in this paper is the first version.

### Functional annotation

For functional annotation, unitigs were used for blastx search against the NCBI non-redundant protein database at E-value 10^–5^. Additionally, we used 27397 protein sequences of *Tribolium castaneum* (considered as model insect), and 20590 of *Acyrthosiphon pisum* (closest insect to *B*. *tabaci*) for Blastx search of unitigs. Top blast hits data was analysed for percent similarity and E-value distribution. The contribution of different species in annotation was also investigated using the top blast hit data of *B*. *tabaci* unitigs against the NCBI nr protein database.

For GO, inter-pro-scan, enzyme code and KEGG pathway analysis, BLAST2GO software was used [20]. Blastx output file (in xml format) was loaded to the BLAST2GO server and used for the above analysis following the standard protocol.

### Gene expression analysis

Illumina sequencing data can be used for accurate estimation of gene expression level by mapping the number of reads for a gene. We calculated the same following the reported protocols [21, 22]. To calculate the gene expression level, we mapped the number of reads to each unitig. Further, the mapped raw read count was adjusted with the length of unitig to calculate reads per kilobase per million mapped reads (RPKM), because the mapping of reads also depend upon the size of reference sequence and amount of sequencing [21].

To further validate the expression of some selected genes of amino acid biosynthesis and transportation pathways, quantitative real time PCR was performed in triplicates on ABI 7500 Real-Time PCR using the SYBR Green PCR Master Mix (Applied Biosystems, CA, USA) following the standard protocol described [23]. *Actin* was used as internal reference gene [24,25] and relative expression was calculated. The reaction mixture (10 μl) consisted of cDNA (0.5 μl), specific primers (5 pmole) (S1 Table) and 2X SYBR Green PCR Master Mix (5 μl), and abundance of transcripts was quantified by ▲▲Ct method [23]. Further, the expression of these genes were also analysed in different developmental stages (egg, nymph and adult) of whitefly.

### Amino acids biosynthesis and transportation

To analyse the genes involved in the biosynthesis of amino acids, unitig sequences were searched for biosynthetic pathway mapping using BLAST2GO and KOBAS server. Unitig sequences, showing significant match to the enzymes involved in the biosynthesis of amino acids, were extracted and analysed for their origin on the basis of similarity to the insect and the symbiont sequences. The genome and 246 protein sequences reported for the symbiont CPA (gene bank accession number NC_018618.1) were downloaded and subsequently utilized for local nucleotide and protein database development.Unitig sequences were used for blast search against the CPA nucleotide and protein databases. Sequences showing significant similarity with the symbiont were further analysed for their contribution in the amino acid biosynthetic pathways using BLAST2GO and KOBAS server. The contribution of the symbiont and the host in amino acid biosynthesis was analysed on the basis of expression of mapped genes to the insect and the symbiont.

The genes encoding for transporters were also identified and used for expression analysis. The sequences for important amino acid transporters reported in aphid [26] were used as database. Unitig sequences were searched against the transporters database for the identification of similar transporters in whitefly at e-value 10^–6^. Top hit sequences with more than 60% similarity and query coverage were considered as putative homologs. Identified amino acid transporters were also used for expression analysis.

## Results and Discussion

### Transcriptome sequencing and functional annotation

The transcriptome sequencing gave a total of 83828866 reads in whitefly, with an average length of 101 bp in a single run on the Illumina sequencing platform. The reads were cleaned by removing the adapter sequences and poly A, T and N sequences. After cleaning 82818787 reads containing 8364697487 bases were assembled into 1324517 scaffolds by ABySS pairwise assembly program [18]. These scaffolds were further assembled by CAP3 program [20]. A total of 34428 contigs and 38288 singletons were pooled together to form 72716 unitigs as distinct sequences of mean size 592 bp for further analysis (Table 1). Length distribution analysis showed 68% unitigs were in the range of 150 to 500 bp. 10823 unitigs with more than 1 kb length were obtained, out of which 201 unitigs were more than 5 kb in length (S1 Fig). These results are in agreement with the earlier report for different species of whitefly [7–9, 27].

**Table 1:** Summary of the transcriptome sequencing and assembly.

Total number of reads	83828866
Total number of clean reads after removing adapter sequence and poly A, T and N sequences	82818787
Average read length	101 bp
Total number of scaffolds after Abyss pairwise assembly	1324517
Total number of distinct sequences (Unitigs) obtained after CAP3 assembly	72716
Longest unitig length	12135 bp
Smallest unitig length	150 bp
Mean length of unitigs	591.9

The accuracy of assembly was confirmed by blastn search of randomly selected unitigs against the NCBI-transcriptome shotgun assembly (TSA) sequences of whitefly. All the tested unitigs showed significant similarity to one or more TSA sequences (S2 Fig). A total of 76.9% (44425/57741) of MEAM1, 81.5% (37365/45831) of Asia II3 and 78% (21309/27288) of MED sequences were mapped with e-value ≤ 10^–5^, that revealed significant homology between these species.

**Fig 1:**
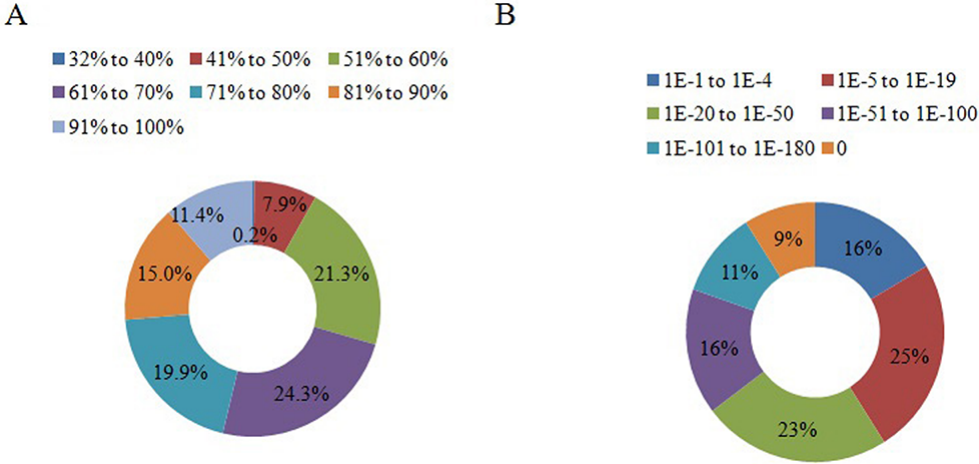
Homology characteristics of whitefly unitigs against the NCBI-nr database. (A) Percent similarity distribution of top blast hits. (B) E-value distribution of top blast hit unitigs.

**Fig 2:**
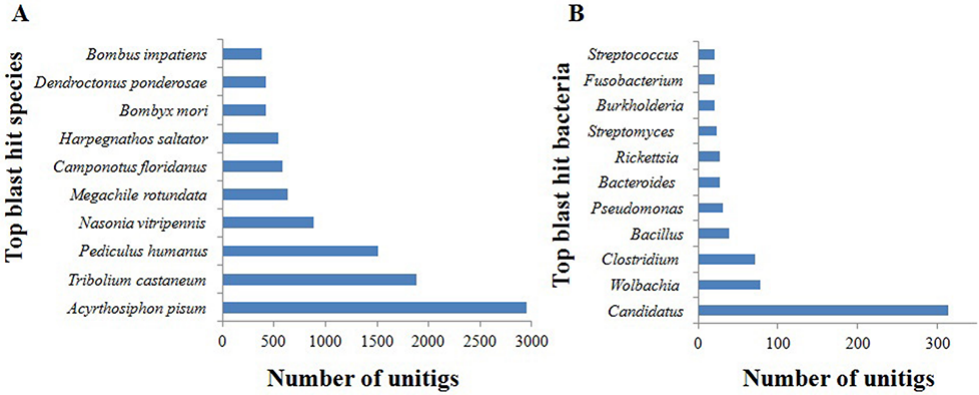
Top blast hit species distribution at NCBI-nr protein database. Top ten blasts hit insect (A) and bacterial (B) species with whitefly unitigs in the NCBI nr protein database.

Blastx search of unitig sequences against the NCBI nr protein database showed significant similarity of 21129 (29%) unitigs with the reported proteins in the database (S2 Table). About 27, 31 and 16% distinct sequences were annotated in case of MEAM1, Asia II3 and MED species, respectively [7–9]. Approximately 70% unitigs were annotated with more than 60% similarity (Fig 1A). E-value distribution of the best hit revealed that ~84% sequences were annotated with e-value smaller than 10^–5^ (Fig 1B). Top hit species distribution analysis showed that the maximum number of sequences matched *Acyrthosiphon pisum* (2954), followed by *Tribolium castaneum* (1880) and *Pediculus humanus* (1509) (Fig 2A; S3 Table). High homology with *A*. *pisum* was expected due to their contiguous taxonomic position (i.e. Homoptera) and similar feeding behaviour.

Whiteflies are known to harbor a number of primary and secondary symbionts, which help in biosynthesis and defence related functions [28,29]. The NCBI nr top blast hit sequences of the transcripts for the mentioned symbionts in the whitefly transcriptome data obtained in the current study were critically analyzed. The *Candidatus* with 313 top blast hit was the most abundant bacteria followed by *Wolbachia* (78) and *Clostridium* (72) (Fig 2B; S4 Table). CPA is reported as the primary endosymbiont of whitefly and is expected to be involved in amino acid biosynthesis in whiteflies [13]. *Wolbachia* and other bacterial species are also reported as symbionts in hemipteran insects [30,31].

### GO annotation and KEGG pathway mapping

**Fig 3:**
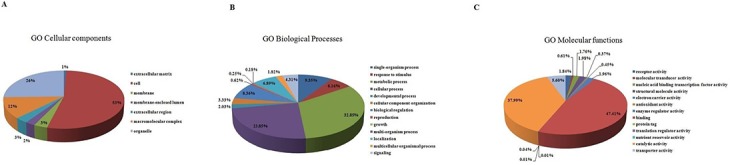
Gene Ontology (GO: level 2) categorization of whitefly unitigs. Sequences were categorized into (A) cellular component, (B) biological process and (C) molecular function on the basis of similarity.

BLAST2GO server was used to assign GO terms to the whitefly unitigs. GO terms have been classified into three categories- cellular components, molecular functions and biological processes. A total of 52847 GO terms were assigned to the 15335 annotated unitigs on the basis of similarity. Out of the total assigned GO terms, 11403 assigned to cellular components, 19890 to biological processes and 24237 to molecular functions (S5 Table). The sum of the GO terms did not match the number of assigned unitigs because several unitigs were classified into more than one group. The number of sequences assigned to GO terms was higher as compared to that in the MED (7330), MEAM1 (4771) and Asia II3 (4819) species [7–9]. This might be due to differences in the amount of sequencing data from each sample. Among cellular components, cell (53%), extracellular matrix (26%) and macromalecular complex (12%) were highly represented (Fig 3A). In case of biological processes, metabolic (32%), cellular (23%) and single organism processes (9%) were most enriched processes (Fig 3B). In the molecular function category, binding (47%) and catalytic (37%) activity were at the top (Fig 3C). About similar representation of GO functional groups is reported for the MED, MEAM1 and Asia II3 species [7–9].

**Fig 4:**
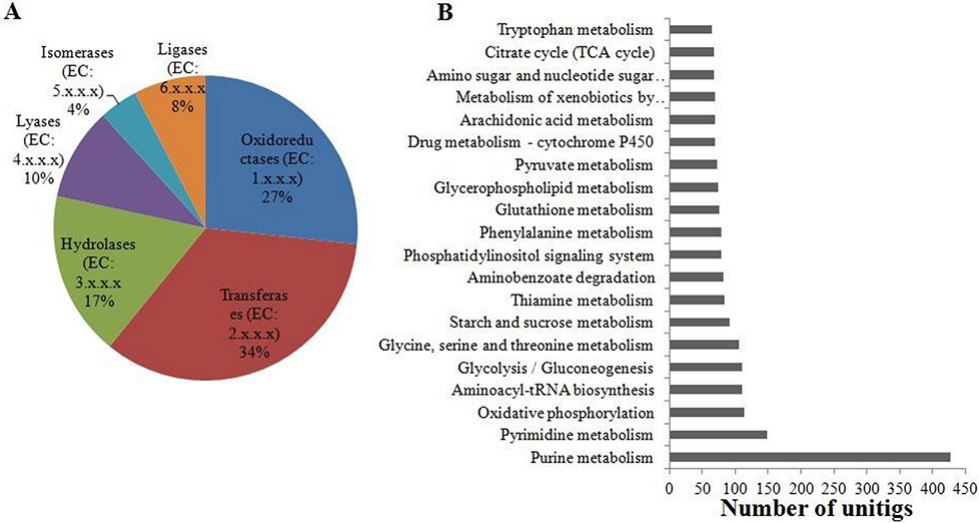
Enzyme classification and KEGG pathway mapping. (A) Percent distribution of different classes of enzymes mapped on whitefly unitigs. (B) Top twenty identified KEGG pathways.

KEGG pathway mapping was also performed by using BLAST2GO server. A total of 4492 annotated unitigs was mapped for 554 enzyme codes and 131 KEGG pathways (S6 Table). Enzyme classification analysis showed that transferases (34%) account for the highest proportion, followed by oxidoreductases (27%) and hydrolases (17%) (Fig 4A). Pathways related to the purine, pyrimidine, amino acid and sugar metabolism were highly enriched. The energy generating pathways like oxidative phosphorylation, glycolysis and pyruvate metabolism were also significantly represented (Fig 4B). Besides these, several pathways involved in drug and xenobiotics metabolism, especially related to the cytochrome P450 were significantly present in the Asia 1, as reported in MEAM 1 and MED species [7,27].

### Gene expression analysis

**Fig 5:**
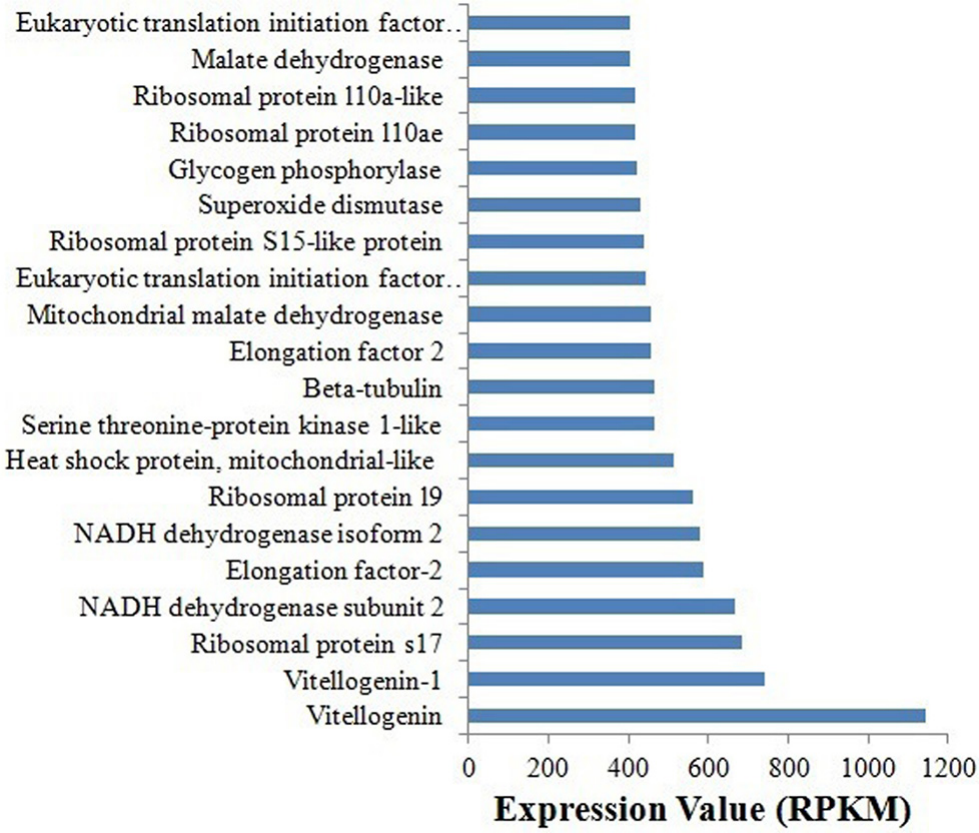
Gene expression analysis of transcriptome data. The figure shows top twenty high expressing genes in whitefly.

The level of gene expression was analyzed by calculating the reads per kilobase per million mapped reads (RPKM) [32]. Summary of the top 100 most expressing transcripts is given in S7 Table. The genes involved in development (vitellogenin), ribosomes (large and small subunits), translation (initiation and elongation factors), cell structure (tubulin) and energy generation (NADH dehydrogenase, malate dehydrogenase) were highly expressed (Fig 5). Similar results are reported for Asia II3 species of *B*. *tabaci* [12]. Since these genes play pivoltal roles in survival and fecundity (vitellogenin), high level of their expression is understandable.

### Amino acid biosynthesis and transporter genes in *B*. *tabaci*

Animals generally lack the genes for the synthesis of essential amino acids, and mostly obtain them from food. Sap sucking insects take their food from phloem sap of the host plant, which contains very little quantity of essential amino acids [10,33–34]. Therefore, these insects often depend on microbial symbionts for the synthesis of these amino acids [35–36]. In return, the host provides nonessential amino acids to the symbiont [12]. Symbiosis of *A*. *pisum* and *B*. *aphidicola* is the best reported model [36–37]. The CPA is reported as a symbiont for whitefly and expected to involve in the biosynthesis of essential amino acids [13]. Genome sequence analysis of CPA clearly indicates that most of the genes responsible for the synthesis of essential amino acids were present in CPA. However, CPA lacks the genes for non-essential amino acid biosynthesis [13]. Since CPA is an endosymbiont, host insect is the only source for the supply of these amino acids.

**Fig 6:**
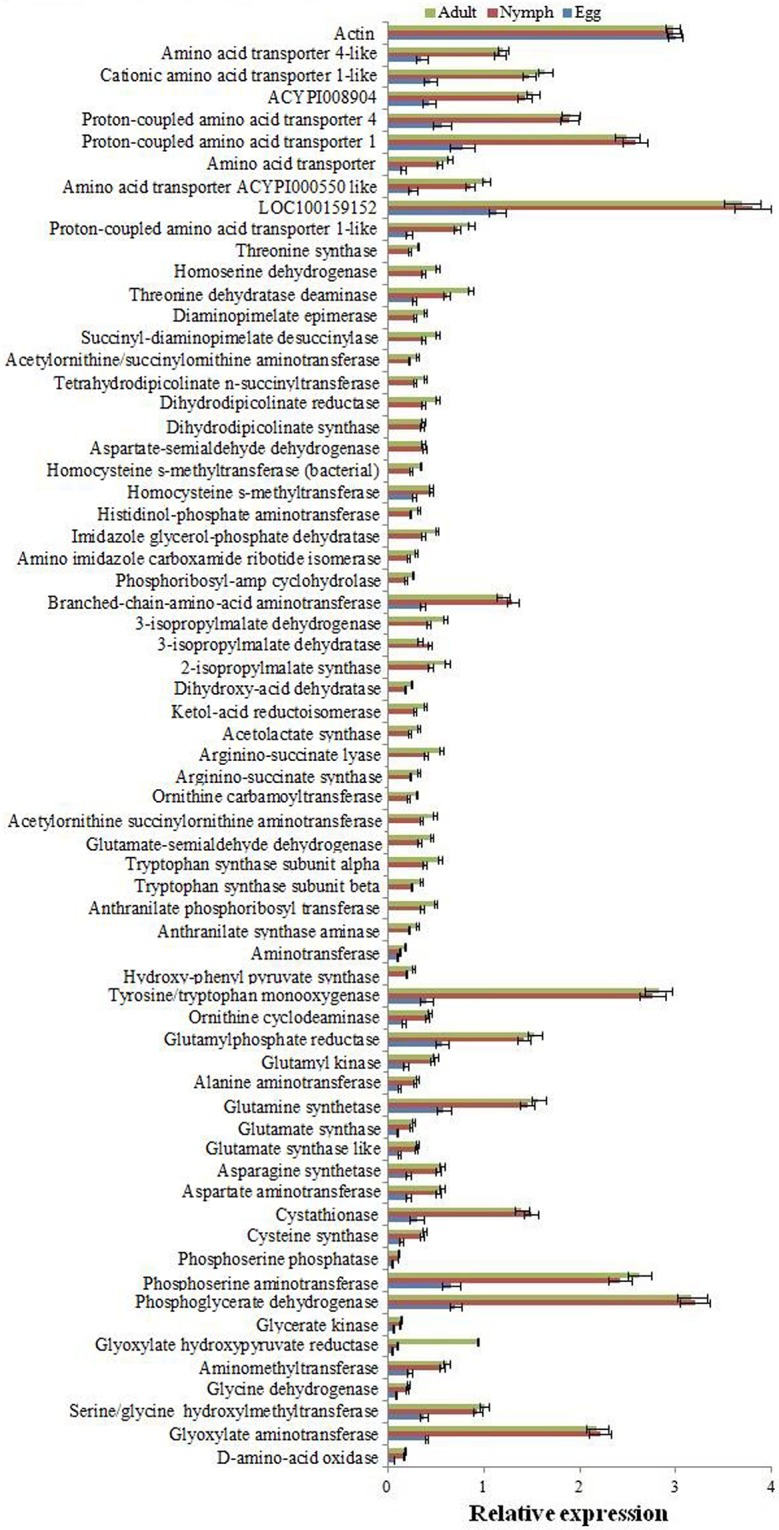
Expression analysis of host and symbiont genes in amino acid biosynthetic pathways. Relative expression analysis of unitigs involved in amino acid biosynthesis in egg, nymph and adult stages of whitefly by quantitative real time PCR. The relative expression of the unitigs in nymph and adult correlates with RPKM value (Table 2) calculated from transcriptome data.

We analysed the interdependence of the whitefly and its symbiont for amino acid biosynthetic pathways by mapping of unitigs for their origin, function and expression (Table 2 and S2, S6, S8 and S9 Tables). Unitigs mapped to the amino acid biosynthetic pathways were analysed for their origin on the basis of similarity with the reported sequences. Unitigs of the symbiont were identified by blastn search against the CPA genome sequence with more than 90% similarity (S9 Table). However, unitigs showing similarity with whiteflies or other insect’s sequences were considered as the hosts’ transcripts. Most of the enzymes involved in the biosynthesis of non-essential amino acids (glutamate, glutamine, aspartate, asparagines, glycines, serine, alanine, cysteine, tyrosine and proline) were encoded by the transcripts of whitefly with reasonably higher expression (Table 2, Fig 6), which is probably sufficient for meeting the requirement of the symbiont also. The result was in agreement with the earlier reports that the CPA lacks the genes responsible for the synthesis of non-essential amino acids [13]. In case of essential amino acid biosynthetic pathways, we observed that the majority of the enzymes was encoded by the symbiont. Since, whitefly lacks the genes for the synthesis of essential amino acids (except threonine and methionine) and phloem sap is also very poor source of these amino acids. The CPA remains the only source to supply these amino acids to whiteflies as *B*. *aphidicola* supplies to aphids [36–37]. We observed that the enzymes responsible for the synthesis of tryptophan and threonine were encoded by the symbiont. Most of the genes involved in the biosynthesis of methionine, lysine, arginine and histidine expressed in the symbiont. However, the complete biosynthetic pathways for these amino acids could not be established from the available transcriptome and genome data [13]. Further, genes involved in the threonine and methionine biosynthesis were detected from the host, also. Since the symbiont is localized in a limited number of cells, the expression value of the genes between whitefly and CPA cannot be compared. Yet, significant expression of most of the genes involved in the synthesis of essential amino acids was noticed in the CPA; which were absent in the whitefly (Table 2). In case of amino acids phenyl-alanine, valine, leucine and isoleucine biosynthesis, CPA lacks the genes encoding enzymes (EC: 2.6.1.1 and 2.6.1.42) for the last step, as also reported earlier in the case of aphid symbionts [11]. However, these genes were available in whitefly with a remarkable expression (Table 2; Fig 6). The results suggested the transport of immediate precursor molecules for the synthesis of these amino acids from symbiont to insect. Our results suggested integration of host whitefly and symbiont CPA genes in amino acid biosynthesis. Both, host and symbiont encoded genes filled the gap in amino acid biosynthetic pathways, and compensate the requirement for each other by synthesizing significant quantity of amino acids as evidenced by high expression of genes.

**Table 2:** The expression value (RPKM) of different genes involves in amino acid biosynthesis in the insect and the symbiont.

	Amino acids	KEGG Pathway map	Enzyme (s)	EC number	Unitig ID	Symbiont RPKM	Insect RPKM
**Non-essential amino acids**	Glycine	260	D-amino-acid oxidase	1.4.3.3	Unitig_2664	-	10.9
			Glyoxylate aminotransferase	2.6.1.44	Unitig_6058	-	147.4
			Serine/glycine hydroxylmethyltransferase	2.1.2.1	Unitig_2506	-	62.2
			Glycine dehydrogenase (decarboxylating)	1.4.4.2	Unitig_61534	-	13.2
			Aminomethyltransferase	2.1.2.10	Unitig_51378	-	37.7
	Serine	260	Glyoxylate hydroxypyruvate reductase	1.1.1.29	Unitig_35285	-	6.7
			Glycerate kinase	2.7.1.31	Unitig_1084	-	8.3
			Phosphoglycerate dehydrogenase	1.1.1.95	Unitig_69094		213.2
			Phosphoserine aminotransferase	2.6.1.52	Unitig_5520	-	161.5
			Phosphoserine phosphatase	3.1.3.3	Unitig_15230	-	6.9
	Cysteine	270	Cysteine synthase	4.2.1.22	Unitig_22283	-	23.3
			Cystathionase	4.4.1.1	Unitig_21860	-	99.6
	Aspartate	250	Aspartate aminotransferase	2.6.1.1	Unitig_64	-	35
	Asparagine	250	Asparagine synthetase	6.3.5.4	Unitig_19156	-	34.6
	Glutamate	250	Glutamate synthase	1.4.1.13	Unitig_47533	-	19.6
			Glutamate synthase	1.4.1.14	Unitig_17057	-	16.1
	Glutamine	250	Glutamine synthetase	6.3.1.2	Unitig_40789	-	96.9
	Alanine	250	Alanine aminotransferase	2.6.1.2	Unitig_16275	-	18.9
	Proline	330	Glutamyl kinase	2.7.2.11	Unitig_19760	-	30.7
			Glutamylphosphate reductase	1.2.1.41	Unitig_69676	-	94.4
			Ornithine cyclodeaminase	4.3.1.12	Unitig_61585		27.2
	Tyrosine	400	Monooxygenase	1.14.16.1	Unitig_52178	-	183.7
**Essential amino acids**	Phenylalanine	400	hydroxyphenylpyruvate synthase	5.4.99.5	Unitig_56082	12.6	-
			prephenate dehydratase	4.2.1.51	Unitig_56082	12.6	-
			Aminotransferase	2.6.1.1	Unitig_21129	-	8.3
	Tryptophan	400	Anthranilate synthase aminase	4.1.3.27	Unitig_42109	14.4	-
			Anthranilate phosphoribosyltransferase	2.4.2.18	Unitig_54016	23.6	-
			Tryptophan synthase	4.2.1.20	Unitig_25708	16.4	-
					Unitig_52877	25.6	-
	Arginine	330	Glutamate-semialdehyde dehydrogenase	1.2.1.38	Unitig_9438	21.8	-
			Acetylornithine succinylornithine aminotransferase	2.6.1.11	Unitig_40277	23.2	-
			Ornithine carbamoyltransferase	2.1.3.3	Unitig_23876	14.3	-
			Argininosuccinate synthase	6.3.4.5	Unitig_24038	15.4	-
			Argininosuccinate lyase	4.3.2.1	Unitig_44282	26.3	-
	Valine, Leucine, Isoleucine	290	Acetolactate synthase	2.2.1.6	Unitig_9069	15.1	-
			Ketol-acid reductoisomerase	1.1.1.86	Unitig_16373	18.3	-
			Dihydroxy-acid dehydratase	4.2.1.9	Unitig_23925	11.6	-
			2-isopropylmalate synthase	2.3.3.13	Unitig_6685	29.4	-
			3-isopropylmalate dehydratase	4.2.1.33	Unitig_6246	29.1	-
			3-isopropylmalate dehydrogenase	1.1.1.85	Unitig_6259	28.4	-
			Branched-chain-amino-acid aminotransferase	2.6.1.42	Unitig_62909	-	86.7
	Histidine	340	Phosphoribosyl-amp cyclohydrolase	3.5.4.19	Unitig_47211	12.3	-
			Phosphoribosylformimino-5-aminoimidazole carboxamide ribotide isomerase	5.3.1.16	Unitig_23937	13.9	-
			Imidazoleglycerol-phosphate dehydratase	4.2.1.19	Unitig_55053	24.2	-
			Histidinol-phosphate aminotransferase	2.6.1.9	Unitig_27079	15.4	-
	Methionine	270	Homocysteine s-methyltransferase	2.1.1.10	Unitig_15630	-	30.1
			Homocysteine s-methyltransferase	2.1.1.14	Unitig_10347	16.2	-
			Histidinol-phosphate aminotransferase	2.6.1.57	Unitig_27079	15.4	-
	Lysine	300	Aspartate-semialdehyde dehydrogenase	1.2.1.11	Unitig_11956	25.2	-
			Dihydrodipicolinate synthase	4.3.3.7	Unitig_56490	23.6	-
			Dihydrodipicolinate reductase	1.17.1.8	Unitig_23804	24.6	-
			Tetrahydrodipicolinate n-succinyltransferase	2.3.1.117	Unitig_11565	18.4	-
			Acetylornithine/succinylornithine aminotransferase	2.6.1.17	Unitig_30242	14.4	-
			Succinyl-diaminopimelate desuccinylase	3.5.1.18	Unitig_11696	24.6	-
			Diaminopimelate epimerase	5.1.1.7	Unitig_39459	18.3	-
	Threonine	260	Threonine dehydratase deaminase	4.3.1.19	Unitig_20690	-	40.9
			Aspartate-semialdehyde dehydrogenase	1.2.1.11	Unitig_11956	25.2	-
			Homoserine dehydrogenase	1.1.1.3	Unitig_9316	24.6	-
			Threonine synthase	4.2.3.1	Unitig_11908	14.8	-

Some non-essential amino acids (like glutamine, glutamate, aspartate and serine) are used as donors of amino groups in the biosynthesis of essential amino acids. Glutamate and glutamine are readily available as a substrate in amino acid and nucleic acid biosynthesis [38]. They act as direct amino group donors in arginine biosynthesis in CPA. Aspartate provides amino group in lysine and threonine biosynthesis [39]. Glutamine and asparagine are reported in high concentration in phloem sap [10], which might also affects the concentration of these amino acids in hemolymph of the phloem feeding insects. Genes involved in the synthesis of these amino acids and for aminotransferase (2.6.1.42) were identified to express at a high level in whitefly (Fig 6; Table 2). The results indicated that the pool of amino group donors for the synthesis of essential amino acids is available in sufficient quantity to compensate the requirement.

**Fig 7:**
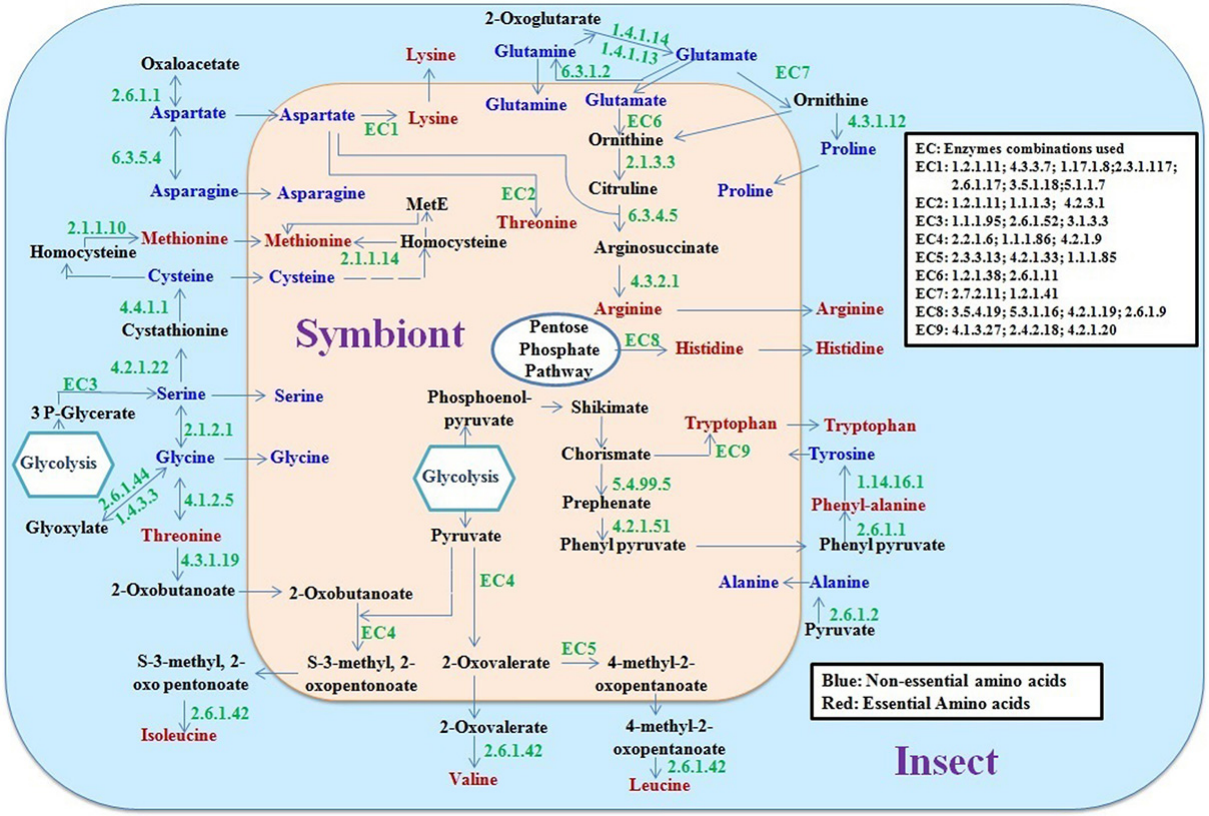
A hypothetical amino acid biosynthetic pathway in whitefly. Pinkish and sky-blue circles represent the symbiont and the insect, respectively. The enzymes are denoted as enzyme commission number (x.x.x.x) in green letters. EC means the enzyme combinations used at particular steps.

The expression of selected genes (Table 2) involved in amino acid biosynthesis was also analysed in different developmental stages of whitefly by quantitative real time PCR analysis in three biological replicates. Significant expression of all the genes was observed in the nymph and adult stages of whitefly, suggesting their production in both the developmental stages (Fig 6). The relative expression pattern was almost similar as calculated from the transcriptome data (Fig 6). However, the expression of genes involved in the essential amino acid biosysthesis (particularly the genes encoded by symbionts) was not detected in the egg (Fig 6). This might be due to either very confined availability of the symbiont and their transcripts in egg; or insect provided enough nutrient to eggs for their development. On the basis of mapping and expression of the symbiont and insect related genes of the amino acid biosynthetic pathways, we predicted a distinct complementing contribution of each partner. A hypothetical model for meeting amino acid needs of the whitefly and its symbiont is given in Fig 7.

Phloem sap is highly deficient in available nitrogen, due to very high carbon/nitrogen ratio. However, nitrogen is important for the growth of insects. Therefore, sap sucking insects developed machinery for the assimilation of nitrogen from ammonia waste. The machinery is well studied in aphid [11], but not reported in whitefly. Genes encoding enzymes glutamate synthase (GltS) and glutamine synthetase (GS) were also detected in whitefly with significant expression (Table 2). The results suggested that whitefly also has the capability to assimilate the ammonia into amino acids as reported in case of aphid [11]. These enzymes are reported to form a shuttle (GOGAT cycle) in plants and microbes for the assimilation of ammonia nitrogen into glutamate [40], which act as substrate for most of the amino acid pathways. GS/GltS is reported as a leading medium for ammonia assimilation in the presence of high glucose concentration [41]. The condition applies to the phloem feeding insects like whitefly and aphids, which feed plenty of sugar and have to maintain low ammonia concentration in cells due to its toxicity. In insects, the GS / GltS cycle is characterized in aphids, mosquitoes and silkworms, where it is associated with ammonia detoxification and nitrogen assimilation in different tissue [11,42–43]. However, the contribution of this cycle in quantitative terms of amino-nitrogen supply is yet not determined.

Transporters play significant role in facilitating cooperation between the host and the symbiont [26]. A total of 628 unitigs were annotated as transporter proteins (S10 Table), in which 71 were the amino acid transporters (S11 Table), which might be involved in transportation of different metabolites and substrate related to the amino acids across the host-symbiont boundary. A total of 34 transporters were considered as high expressing (RPKM>50), which include amino acid transporter, proton-coupled amino acid, sodium nucleoside, sugar, trehalose, organic cation, monocarboxylate and ABC transporters. Several types of amino acid transporters like proton-coupled, neutral and basic amino acid, cationic amino acid and sodium-dependent neutral amino acid were detected with high levels of expression. Besides these, the expression of several specific transporters like glutamate, tyrosine transporter and vacuolar amino acid transporter were also detected. Similar transporters are highly expressing in case of aphids [11,44]. Cationic amino acid transporters involve in arginine, lysine and histidine transport, however, branched-chain amino acid transporters facilitate the transport of phenyl-alanine, valine, leucine and iso-leucine [45]. The excitatory amino acid transporter plays significant role in glutamate transportation (http://www.sdbonline.org/sites/fly/genebrief/eaat1.htm).

Forty putative amino acid transporters are reported recently in *A*. *pisum*; five of them (ACYPI000536, ACYPI000550, ACYPI001018, ACYPI008904 and ACYPI008971) are reported as more imperative in host-symbiont interaction [26,45]. Among these, two (ACYPI001018 and ACYPI008904) genes are involved in glutamine transport. We also identified putative homologs of these five transporters in whitefly with significantly higher expression (Table 3). The expression of these transporters was further confirmed by quantitative real time PCR in triplicates (Fig 6). The results suggested that whitefly expresses the amino acid biosynthesis pathways and the transporters for sharing these across the host symbiont boundary. Since glutamine is synthesized in the insect and is an important source of amino group in the biosynthesis of several essential amino acids in the symbiont; its transport in significant quantity across the host-symbiont boundary is reported [11]. Two (ACYPI001018 and ACYPI008904) transporters are reported to be actively involved in glutamine transport in aphids, in which ACYPI001018 is the dominant transporter followed by ACYPI008904 [26]. Availability of genes encoding these transporters with high expression indicated similar mechanism of cooperation between whitefly and CPA as reported for aphids and *Buchnera* [26]. However, the exact role of these transporters in whitefly needs to be validated in future studies by following either recombinant expression or silencing or both the approaches.

**Table 3:** Expression value of the putative homologous unitigs in whitefly for the top five amino acid transporters of aphid.

Amino acid transporters of aphid (Price et al. 2014)	Putative homologous unitigs of whitefly	Expression value (RPKM)
ACYPI000536 (LOC100159138)	Unitig_36850	48
ACYPI000550 (LOC100159152)	Unitig_68785	253
	Unitig_21655	57
	Unitig_40249	36
ACYPI001018(LOC100159667)	Unitig_47082	172
	Unitig_49482	126
ACYPI008904 (LOC100168178)	Unitig_52040	95
	Unitig_52329	98
ACYPI008971 (LOC100168251)	Unitig_36271	78

## Conclusions

Comprehensive analysis of the transcriptome data of the whitefly has provided important information regarding the mechanism of cooperation between the host and its symbiont by sharing distinct amino acid pools. Whitefly feeds on phloem sap which contains low levels of essential amino acid and lacks its own gene for their synthesis. Whitefly contains genes for the synthesis of non-essential amino acids and expresses those at significantly high level. On the other hand, the symbiont CPA produces essential amino acids by sharing some metabolites with the host insect, but lacks the genes for non-essential amino acids. These results along with the significant expression of several amino acid transporters suggest various levels of cooperation between the host and the symbiont. Future studies at the protein and biochemical level will define subsequent details of interdependence between the two partners in the symbiosis.

## Supporting Information

S1 Fig Length distribution analysis of whitefly unitigs. Digit on the top of each bar gives the number of unitigs in each range.
(JPG)

S2 Fig Blast analysis of selected unitigs. Colour key for blast alignment score of selected unitigs against NCBI-transcriptome shotgun assembly (TSA) sequences of *B*. *tabaci*.
(JPG)

S1 Table List of primers. Primers used for the quantitative real time PCR amplifications.
(XLSX)

S2 Table Annotation of *B*. *tabaci* unitigs. Annotation details of *B*. *tabaci* unitigs with the NCBI nr protein database using BlastX.
(XLSX)

S3 Table Top blast hit species. Top hit species distribution of *B*. *tabaci* unitigs against the NCBI nr protein database.
(XLSX)

S4 Table Top blast hit bacterial species. Top hits bacterial species in NCBI nr protein database.
(XLSX)

S5 Table GO analysis. GO functional annotation details of *B*. *tabaci* unitigs.
(XLSX)

S6 Table KEGG analysis. Details of KEGG pathways identified.
(XLSX)

S7 Table Most abundant transcripts in *B*. *tabaci*. Summary of the top 100 most abundant transcripts of *B*. *tabaci*.
(XLSX)

S8 Table Amino acid biosynthesis pathways. Details of amino acid biosynthesis pathways identified in *Candidatus Portiera aleyrodidarum*.
(XLSX)

S9 Table Blastn for symbiont gene search. Identification of symbiont “*Candidatus Portiera aleyrodidarum”* unitigs by blastn search against the genome sequence (accession number NC_018618.1).
(XLSX)

S10 Table Transporter proteins. List of unitigs annotated as transporter proteins.
(XLSX)

S11 Table Expression of transporters. List of amino acid transporters and their expression value in *B*. *tabaci*.
(XLSX)
