# Supplementary figures and images for: Whitefly Genome Expression Reveals Host-Symbiont Interaction in Amino Acid Biosynthesis
Source: PLoS One. 2015 May 22;10(5):e0126751. doi: 10.1371/journal.pone.0126751 (PMC4441466; doi:10.1371/journal.pone.0126751)

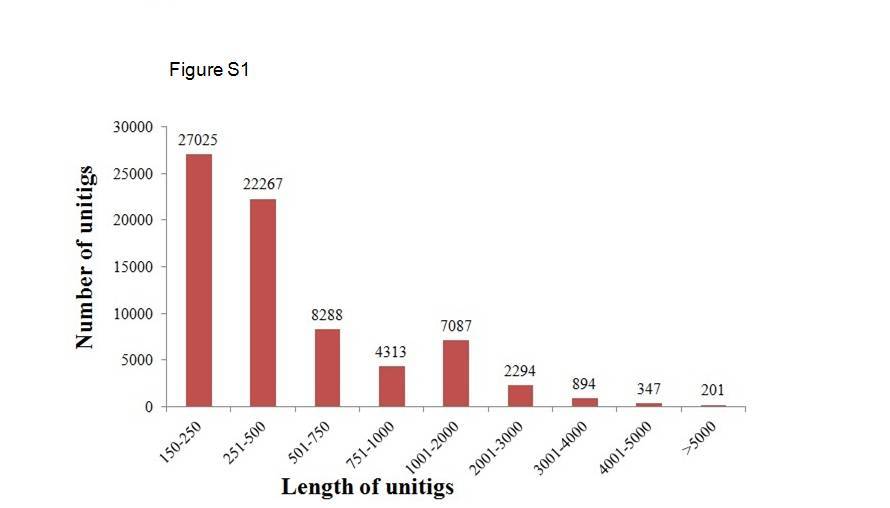

Supplement: S1 Fig — Digit on the top of each bar gives the number of unitigs in each range. (JPG) [file pone.0126751.s001.jpg]

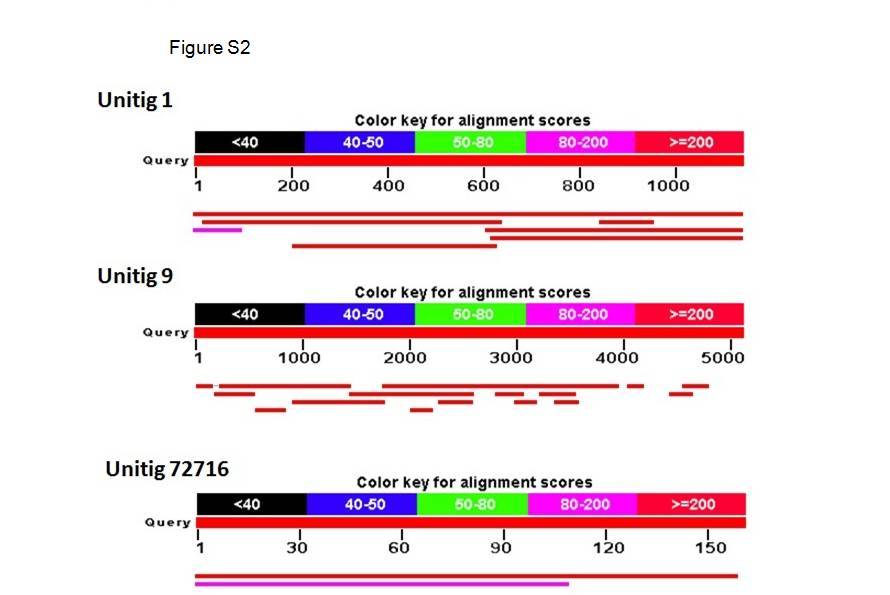

Supplement: S2 Fig — Colour key for blast alignment score of selected unitigs against NCBI-transcriptome shotgun assembly (TSA) sequences of B. tabaci. (JPG) [file pone.0126751.s002.jpg]
